# Doctors’ Personal Preference and Adoption of Mobile Apps to Communicate with Patients in China: Qualitative Study

**DOI:** 10.2196/49040

**Published:** 2024-06-10

**Authors:** Dongjin Chen, Wenchao Han, Yili Yang, Jay Pan

**Affiliations:** 1 Institute for Social Governance and Communication Innovation of Zhejiang Communication University of Zhejiang Hangzhou China; 2 Center for Asia-Europe Study Xi'an Jiaotong University Xi’an China; 3 HEOA Group, Institute for Healthy Cities and West China Research Center for Rural Health Development Sichuan University Chengdu China; 4 West China School of Public Health and West China Fourth Hospital Sichuan University Chengdu China

**Keywords:** medical platforms, doctor-patient communication, social networking apps, thematic content analysis, China

## Abstract

**Background:**

Different kinds of mobile apps are used to promote communications between patients and doctors. Studies have investigated patients’ mobile app adoption behavior; however, they offer limited insights into doctors’ personal preferences among a variety of choices of mobile apps.

**Objective:**

This study aimed to investigate the nuanced adoption behaviors among doctors in China, which has a robust adoption of mobile apps in health care, and to explore the constraints influencing their selection of specific mobile apps. This paper addressed 3 research questions: (1) Which doctors opt to adopt mobile apps to communicate with patients? (2) What types of mobile apps do they choose? (3) To what degree do they exercise personal choice in adopting specific mobile apps?

**Methods:**

We used thematic content analysis of qualitative data gathered from semistructured interviews with 11 doctors in Hangzhou, which has been recognized for its advanced adoption of mobile technology in social services, including health care services. The selection of participants was purposive, encompassing diverse departments and hospitals.

**Results:**

In total, 5 themes emerged from the data analysis. First, the interviewees had a variety of options for communicating with patients via mobile apps, with the predominant ones being social networking apps (eg, WeChat) and medical platforms (eg, Haodf). Second, all interviewees used WeChat to facilitate communication with patients, although their willingness to share personal accounts varied (they are more likely to share with trusty intermediaries). Third, fewer than half of the doctors adopted medical platforms, and they were all from tertiary hospitals. Fourth, the preferences for in-person, WeChat, or medical platform communication reflected the interviewees’ perceptions of different patient cohorts. Lastly, the selection of a particular kind of mobile app was significantly influenced by the doctors’ affiliation with hospitals, driven by their professional obligations to fulfill multiple tasks assigned by the hospitals or the necessity of maintaining social connections with their colleagues.

**Conclusions:**

Our findings contribute to a nuanced understanding of doctors’ adoption behavior regarding specific types of mobile apps for patient communication, instead of addressing such adoption behavior of a wide range of mobile apps as equal. Their choices of a particular kind of app were positioned within a social context where health care policies (eg, limited funding for public hospitals, dominance of public health care institutions, and absence of robust referral systems) and traditional culture (eg, trust based on social connections) largely shape their behavioral patterns.

## Introduction

The widespread usage of smartphones, embraced by over 6 billion individuals, has facilitated the proliferation of mobile apps among users worldwide. Within this landscape, the integration of mobile apps to facilitate online medical services has emerged as one of the most rapidly evolving sectors in the health care industry. The term “mobile health”, or “mHealth,” is defined by the World Health Organization (WHO) as “medical and public health practice supported by mobile devices, such as mobile phones, patient monitoring devices, Personal Digital Assistants (PDAs), and other wireless devices” [1]. In addition to mHealth, eHealth [2], telehealth [3], and other newly emerged terms have also been used to describe the dynamic evolution of contemporary online health services. As demonstrated by numerous recent studies, the adoption of mobile apps in health services has immense potential to enhance doctor-patient communications, primarily through 2 types of mobile apps: online medical platforms and social networking apps. In this context, exploring the functioning mechanisms of these mobile apps has become imperative for identifying barriers to mHealth penetration, thereby offering insights to inform the targeted expansion of specific app types to improve the quality and efficiency of health care delivery procedures.

In China, more than 1 billion people used mobile phones in 2022 [4]. As the stressful relationship between health care providers and patients persists as a longstanding issue in the context of China’s health care system [5], the adoption of mobile apps presents a promising strategy to mitigate this tension to some extent [6]. Recent studies have highlighted the benefits conferred by mobile apps, including promoting health care delivery procedures regardless of geographical barriers [7], contributing to the alleviation of urban-rural health care disparities by offering online medical consultation to those living in remote areas [8], and expanding the range of service areas for health care professionals who provide quality specialized care via mobile connections [9]. As such, the adoption of mobile apps proves to be a vital strategy for advancing both health care delivery and the doctor-patient relationship.

The adoption of mobile apps has brought numerous benefits, yet scholars have raised concerns regarding various issues encountered during implementation. Among these concerns, protecting patients’ online personal information stands out as a significant security challenge [10]. In addition, health care professionals’ use of social media apps might lead to an identity crisis, blurring the boundaries between their personal and professional lives [11]. Most studies have emphasized individual-level factors as major determinants of personal preference, yielding diverse findings on aspects such as social and economic returns [12], extrinsic and intrinsic motivations [13], their disciplines [7], their professional titles [14], anticipated rewards [15], and monetary incentives [13].

Despite different findings and conclusions, these studies were based on an implicit assumption that these doctors have full control of their choices, which means that doctors have the freedom to decide how to spend their spare time at their own will. However, in China, this assumption warrants scrutiny due to the well-known issue of excessive workload faced by health care professionals, leaving them with limited leisure time after extended hours [16].

Previous studies have identified the level of doctors’ affiliated health care organizations as a potential factor that affects the adoption of web-based health information technologies. For example, Li et al [14] found that quality doctors from tertiary hospitals exhibit greater activity on web-based medical consultation platforms. However, the underlying mechanism of this influence requires further explanation, not to mention that some macrolevel influences remain to be explored, such as the impact of national health care policies. These influences are beyond doctors’ personal choices. Nevertheless, a few exceptions from the current literature managed to analyze meso- and macrolevel factors, in addition to individual-level factors. Peng et al [17] applied social ecosystem theory to understand the determinants of physicians’ online uptake and regarded their perceptions of law and regulation of online health as macrolevel factors. However, these meso- and macrolevel factors proposed for analysis primarily pertain to online health care services, thus neglecting the potential impacts of the particular contexts. Other scholars have conducted review studies describing the current status of China’s mHealth industry, with emphasis of the overall impacts of the health care system, but have not provided details of the underlying mechanism [6].

To address the existing gap in the current literature, this study aimed to address the nuanced mobile app adoption behaviors of doctors in China and explore the factors influencing their choice of specific mobile apps, rather than merely examining general adoption behavior. We conducted thematic analysis of semistructured interviews with selected doctors in Hangzhou, focusing on these 3 questions: (1) Which doctors opt to adopt mobile apps to communicate with patients? (2) What type of mobile apps do they adopt? (3) To what degree do they exercise personal choice in adopting specific mobile apps?

## Methods

### Research Design

The inductive approach was used to facilitate exploratory research, and interviews were conducted to understand interviewees’ inner thoughts about the issue being investigated [18]. Thematic content analysis was then used to uncover subtle concerns underlying their adoption behavior. This qualitative method has been used to explore health care services provision from the supply side in order to understand health care professionals’ perception of internet hospitals [19], as well as their use of mHealth in practice to deliver health care services [20]. Thus, the self-reported narratives from interviewees provided direct responses to the set of research questions we previously proposed.

### Study Setting

We conducted our study in Hangzhou, the provincial capital of Zhejiang Province. Hangzhou City is situated on the east coast of China, approximately a 2-hours ride from Shanghai. We selected this city as the research setting mainly out of 3 considerations. First, with a population of 12.20 million and a local gross domestic product (GDP) per capita of Renminbi (RMB) 149,900 (~US $21,122) [21], significantly higher than the national average of RMB 80,000 (~US $11,273) in 2021 [22], the city has a relatively affluent population. This means local residents are more likely to use their out-of-pocket money to pay the fee charged by medical platforms. Second, this city is renowned for its technical advancement and has attracted a lot of mobile app companies (eg, Taobao, the biggest online shopping company in China) to build their headquarters there. Third, this city is home to 3 hospitals ranked among China’s top 50 hospitals, where highly skilled health care professionals are more likely to embrace advanced health information technology [23]. Therefore, this city presents an ideal research environment for investigating the current landscape of mobile app adoption in China’s clinical settings and exploring the issues and concerns perceived by health care professionals during their practical interactions with mobile apps.

### Data Collection

We conducted purposeful sampling to select participants for in-depth interviews. Given that investigating doctors’ personal choice of mobile apps might involve disclosure of their or their patients’ private information, securing cooperation from the doctors proved challenging.

We selected participants using the following criteria: First, they were under 50 years old, as older individuals are less likely to adopt new technology. Second, we tried to cover different levels of medical agencies, including local health community clinics and tertiary hospitals. Considering that doctors working in tertiary hospitals are more likely to use mobile apps to provide online medical consultation services [14], more than half of the interviewees were selected from tertiary hospitals. Third, we tried to cover doctors specializing in different areas, as well as with different professional titles. Fourth, as most medical agencies in China are public agencies, we selected our interviewees primarily from public hospitals.

### Interviews

Prior to interviews, the researchers sent the topic list to the interviewees so that they could prepare for the interviews ahead. All 11 interviews occurred within hospital settings, with the interviewers maintaining impartiality. Four of the researchers conducted the interviews, two of whom (authors DC and JP) possessed previous research experience with interviewees, while the other two (authors WH and YY) received training in interview skills. Field notes were taken during interviews, and audio recordings were made with the interviewees’ consent. Each interview commenced with a brief introduction of the interviewer’s research background and the research project. The interviewees were given the option to decline participation, although nobody dropped out. We conducted the interviews from June to December 2022, and each interview lasted for approximately 30-60 minutes. Interviewees received RMB 200-400 (~US $27-55), depending on the interview duration. Data saturation was achieved after interviewing 11 doctors.

### Primary Model and Interview Questions

**Figure 1:**
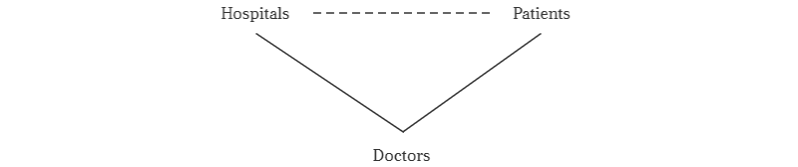
The relationship triangle between doctors, patients, and hospitals.

Different from previous studies focusing on a straight relationship between doctors and patients, we added another bilateral relationship between doctors and hospitals to our analyses, forming a basic framework to approach this research. Current studies have underscored the interconnectedness between offline and online health care services [15,24]. It is necessary to investigate institutional impacts posed by their affiliated hospitals, which are also the employers of their offline practice. We devised a triangular model to understand the dynamics between doctors, patients, and hospitals, as depicted in Figure 1. This model delineates 3 sets of dual relationships: doctor-patient, doctor-hospital, and patient-hospital. We constructed our interview questions based on this model and collected interviewees’ responses to each question accordingly. The interview questions are listed in Multimedia Appendix 1.

### Data Analysis

Two researchers (DC and WH) transcribed all the recorded files and reviewed them for accuracy. According to Braun and Clarke [25], both manual coding and software coding are applicable for thematic analysis. Drawing from the interviewees’ spoken words, we used manual open coding to identify potential themes. The analysis included both inductive and deductive coding [26]. The primary model outlined in Figure 1 guided deductive coding, while transcribed data yielded unanticipated themes, necessitating inductive coding. The two researchers (DC and WH) read through the interview transcripts and applied both inductive coding and deductive coding to generate an individual code book independently. Next, they cross-referenced their respective code book, and any discrepancies were resolved through discussion with a third researcher (JP), ultimately reaching consensus on overarching themes. Following 11 interviews, no new themes emerged and saturation was reached. The researchers adhered to the COREQ (Consolidated Criteria for Reporting Qualitative Research) checklist to report the empirical research process [27].

### Ethical Considerations

Ethical approval for this research was obtained from News and Communication Research Institute, Communication University of Zhejiang. Prior to interviews, the researchers assured the interviewees of the anonymization of their personal information, and all interviewees provided written informed consent.

## Results

### Research Process

**Figure 2:**
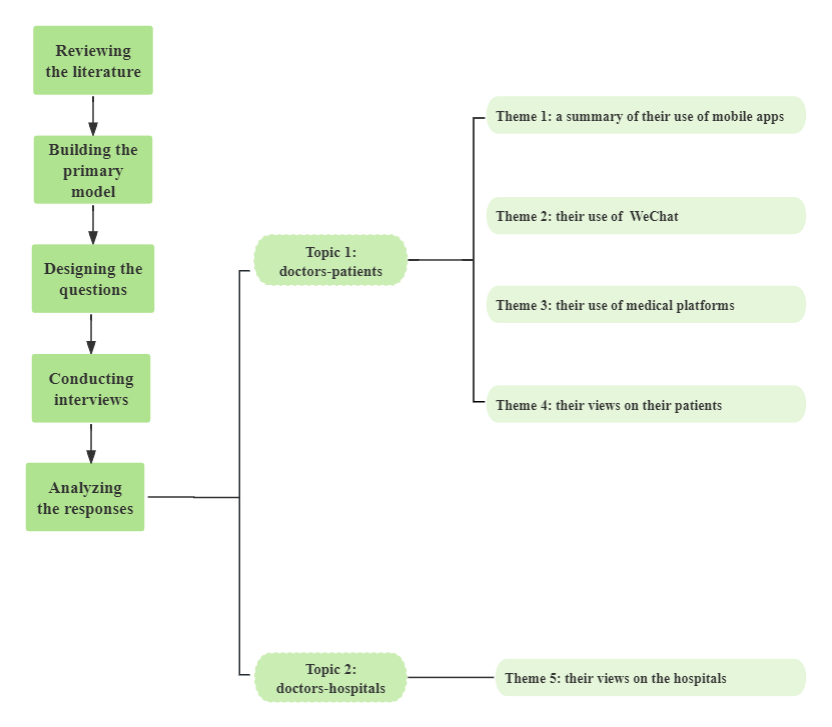
Stages of the research process.

Each step of the research process is presented in Figure 2. We extracted 5 themes from the data analysis, including a summary of the interviewees’ mobile app usage, the specific use of 2 primary kinds of mobile apps, and the interviewees’ views on patients and affiliated hospitals.

### Summary of Interviewees’ Use of Apps

We interviewed a total of 11 participants, 9 (82%) of whom were from tertiary hospitals, with 2 (18%) from top-tier hospitals in China, ranked among the top 50 hospitals. Table 1 delineates the key characteristics of each interviewee. We added 1 category to the table to describe the varying degrees of difficulty in making an appointment with these doctors. Specifically, “very difficult” means that a waiting period of approximately 1 month is necessary for potential patients to compete for a possible slot either over the phone or through the online registration system, while “difficult” suggests a shorter waiting time of 1-2 weeks. For the remaining doctors, no appointment is required in advance, as they provide readily accessible services to patients on their clinical days.

**Table 1:** Characteristics of interviewees (N=11) collected from June to December 2022.

Characteristics		Interviewees, n (%)
**Gender**		
	Male	8 (73)
	Female	3 (27)
**Age (years)**		
	30-40	3 (27)
	40-50	8 (73)
**Professional title**		
	Chief physician	2 (18)
	Associate chief physician	5 (45)
	Attending doctor	4 (36)
**Type of affiliated hospital**		
	Public tertiary hospital	9 (82)
	Community clinic	1 (9)
	Private clinic	1 (9)
**Difficulty in making an appointment**		
	Very difficult	3 (28)
	Difficult	4 (36)
	Not difficult	4 (36)
**Department director or not**		
	Department director	3 (27)
	Not department director	8 (73)
**Affiliated department**		
	Surgery	2 (18)
	Internal medicine	2 (18)
	Traditional Chinese medicine	4 (36)
	Gynecology	1 (9)
	Dentistry	2 (18)

According to the interviewees, they used a variety of mobile apps for patient communication, with the predominant ones being social networking apps (eg, WeChat) and medical platforms (eg, Haodf). All interviewees used WeChat to communicate with patients, either through 1-to-1 chats or group chats, although their willingness to share their personal WeChat accounts with patients varied. Only 4 (36%) interviewees reported using medical platforms at the time of the interviews, with detailed use discussed in later sections.

In terms of communication style (the number of people engaged in the communication), 1-to-1 chats via WeChat involve 2 people. WeChat-facilitated group chats entail the participation of multiple individuals, commonly including 1 or several doctors, alongside a group of patients. These discussions typically focus on medical consultation for specific conditions or the dissemination of supportive information, such as guidance on appointment scheduling. The medical platforms enable doctors to have 1-to-1 conversations with anonymous patients, and these conversations or the feedback on them can be further reviewed by other end users whenever they access the same medical platforms. Meanwhile, patients’ medical examination results and their identification information remain confidential.

### The Use of WeChat

WeChat is the most widely embraced social media app in China. In addition to text-based messages, it supports multimedia communication through the sharing of images, recordings, and videos, alongside the integration of numerous miniapps. Table 2 presents a summary of the interviewees’ use of WeChat to communicate with patients or potential patients. Notably, 1-to-1 chats were the most personalized and confidential mode of communication, involving interactions between 2 individuals.

**Table 2:** Interviewees’ use of specific WeChat functions to communicate with patients from June to December 2022.

Interviewee ID	Specific WeChat function			
	One-to-one chat^a^	Group chat	WeChat Moments (friend circle)	WeChat short-term video
1	Likely	—^b^	Yes	—
2	Rare	—	—	—
3	Very likely	—	Yes	Yes
4	Very likely	Yes	Yes	Yes
5	Rare	—	—	—
6	Very likely	Yes	—	—
7	Likely	—	—	—
8	Rare	—	Yes	Yes
9	Rare	—	Yes	—
10	Likely	Yes	—	—
11	Very likely	—	Yes	—

^a^The frequency of 1-to-1 chats was measured based on the average number of patients with whom these interviewees had WeChat conversations in 1 week: <5 patients measured as “rare”; 6-8 patients as “likely”; ≥9 patients as “very likely”.

^b^Not applicable.

Of the 11 doctors, 4 (36%) exhibited a propensity to share their personal WeChat accounts with patients very likely; these doctors were all from low-risk departments, including internal medicine, dentistry, or traditional Chinese medicine. In contrast, 4 (36%) doctors rarely did so due to safety concerns with potential patients.

Yes, I will share my WeChat account with patients. After I joined this private dental clinic, my patients were first allocated by the clinic. Later, as these patients found satisfaction with my services, they began to introduce my account to other people, and I have more and more patients.

Interviewee 3

Additionally, a few doctors adopted a discerning approach, depending on initial interactions with newly admitted patients, regarding assessments of medical compliance as a determining factor.

I will make an evaluation of patients when deciding to share my WeChat account with them. I select those patients who are reasonable and cooperative, with a good sense of medical compliance. I am very cautious against those picky patients. Usually, doctors do not have obligations to share their personal account with patients. We do so only for the sake of their conveniences.

Interviewee 10

Furthermore, all interviewees expressed a willingness to share their personal accounts with those introduced by well-acquainted intermediaries.

I will not share my WeChat account with patients. The rare exceptions are for some special patients, depending on whether patients’ parents are easy to communicate with and their diseases need long-time follow-up. In addition, I will share my account with those who are introduced by my familiar colleagues, friends, and relatives. China is a society replete with social connections, and I cannot avoid these connections.

Interviewee 8

Group discussions are another frequently used communication form on the WeChat platform, especially for patients with chronic diseases requiring long-term care and occasional relapse. Some WeChat groups can be created by institutions, hospitals, or departments. One local clinic (the employer of interviewee 10) created WeChat groups including 2 or 3 family doctors to meet the needs of local residents. Alternatively, interviewees may initiate their own groups. Here is an interviewee’s response.

In my WeChat account, I have ten WeChat patient groups. Each group has 500 patients. Those initially added to the group invited their friends and relatives, and the number of each WeChat group reached the maximum capacity of 500 very quickly.

Interviewee 6

Doctors’ adoption of WeChat facilitates effective knowledge dissemination in the form of photos, articles, and short video demonstrations (eg, WeChat Moments and WeChat short-term video-sharing functions). Some doctors, such as a department chair (interviewee 8) from a newly established department, demonstrate high levels of proactivity in using video-sharing functions to promote their reputation in order to attract new patients.

### The Use of Medical Platforms

In China, medical platforms serve multiple functions, primarily facilitating medical appointments, medical consultations, and online prescriptions. Except an interviewee from a private dental clinic, all remaining interviewees’ affiliated hospitals have already offered online platforms to execute some basic functions. These hospitals have created their own WeChat official accounts to provide supporting services via integrated miniapps in WeChat, such as making medical appointments and checking medical exam results, or created their own medical platforms to provide a wider range of services. Table 3 outlines the hospitals’ involvement in mHealth, enhancing patients’ convenience in scheduling appointments via mobile apps. However, considering this kind of functionality does not necessitate interactions between doctors and patients, our investigation primarily focused on functionalities reliant on patient-doctor interactions on medical platforms.

**Table 3:** Affiliated hospitals’ involvement in mHealth^a^ adoption from June to December 2022.

Involvement of interviewees’ employers in mHealth	Specific functions	Interviewees (N=11), n (%)
mHealth functions built into the hospitals’ official WeChat account	Making medical appointments Reviewing medical exam results	10 (91)^b^
Their own medical platform exclusively designed for 1 hospital	Making medical appointments Reviewing medical exam results Preparing prescriptions Having online consultations with both first-time and follow-up patients	1 (9); interviewee 5
Official partnering with a medical platform designed for many hospitals	Making medical appointments Reviewing medical exam results Preparing prescriptions Having online consultations with both first-time and follow-up patients	1 (9); interviewee 8
No mHealth app	N/A^c^	1 (9); interviewee 3^d^

^a^mHealth: mobile health.

^b^Although these interviewees’ affiliated hospitals were involved in mHealth to varying degrees via official WeChat accounts, the interviewees were not involved, since there was no need to communicate with patients via accounts at the time of the interviews.

^c^N/A: not applicable.

^d^The interviewee was from a private dental clinic.

Based on our findings, interviewees’ use of medical platforms to communicate with patients was not as frequent as their use of WeChat. Of the 11 doctors, only 4 (36%) were using medical platforms at the time of the interviews, including Haodf (the largest commercial medical platform in China), Nali (a platform having an official partnership with some hospitals), the affiliated hospital’s official platform, and Gancao Doctor (a commercial platform focusing on traditional Chinese medicine). They were all from tertiary hospitals. Of these 4 doctors, 2 (50%) were from esteemed top-tier hospitals (interviewees 5 and 8) and the other 2 (50%) from average tertiary hospitals (interviewees 6 and 9). They used multiple medical platforms simultaneously, with Haodf being the most popular.

The motivations behind interviewees’ choices of specific platforms varied. Building departmental reputation (interviewees 8 and 9) and providing more convenience to offline patients (interviewees 5, 6, and 9) were reported as 2 key incentives for interviewees who need to perform surgeries. These interviewees have too many offline patients, which makes it rather difficult for some of those patients to make a timely appointment in the offline service. As admitted by these interviewees, the volume of patients using medical platforms is quite limited compared to their offline patients, and some of the online patients are diverted from offline services to seek more further suggestions.

One department director (interviewee 9) highlighted that using a medical platform could enable doctors to earn an extra income, and encourages subordinates, particularly young doctors, to use medical platforms to provide medical consultations to patients and then to attract more online patients to offline services. A young attending doctor (interviewee 6) reported actively using a medical platform (Gancao Doctor) to prescribe traditional Chinese medicine to patients. As explained by this doctor, following the initial in-person medical consultation that occurs at the hospital, subsequent follow-ups can be conducted online, which significantly enhances the convenience for those living in remote areas and generates an additional income for the doctor.

Some interviewees had different reasons for not using medical platforms. Interviewees 1 and 4 had tried medical platforms but ultimately discontinued their usage due to the limited number of patients engaged on those platforms. Some interviewees were overwhelmed by their offline duties and, thus, were not willing to further sacrifice their precious spare time (interviewees 1-3 and 7). Interviewee 10 from a local clinic was not busy but was still reluctant to use mobile apps related to work beyond working hours, due to family obligations. Interviewees 3 and 11 regarded WeChat as an effective approach to maintaining their connections with their patients, and they perceived WeChat as a more convenient alternative to medical platforms.

Cross-comparing the interviewees’ responses on whether use medical platforms presented a contradictory understanding of the possible influence of heavy workloads from offline services. A heavy workload can either discourage or encourage the adoption of a medical platform. A cross-sectional study found no statistically significant association between workloads and doctors’ uptake of e-hospitals [28]. Our interviewees’ responses highlighted the underlying reason. Their different choices reflected their views on how to maintain a balance between professional duties and personal life. More than half of the interviewees from average tertiary hospitals preferred focusing solely on offline patients to preserve some personal time. Conversely, both interviewees from esteemed top-tier hospitals were extremely busy, leaving little opportunity to prioritize their personal life during spare moments. Despite these divergent choices, interviewees from tertiary hospitals unanimously agreed that personal time is severely limited.

### Interviewees’ Views on Patients

The interviewees had a mixed view on the strengths and weaknesses of different communication methods, and they all preferred face-to-face communication. In addition to more comprehensive examinations of patients’ symptoms, they contended that such in-person communication would facilitate the detection of subtle signs and symptoms with greater precision. Another concern arising from online conversations was the heightened cautiousness that doctors exhibit during conversations, as indicated by interviewee 8’s response. The reason is that all conversations are documented in written form and might serve as evidence in the case of patient complaints. However, they admitted that the strength of online mobile communication lies in its convenience to patients, such as enabling them to review their medical exams results and providing follow-up suggestions.

I have many years’ working experience, and I have a strong sense of cautiousness when dealing with online communication risks. I will add some preconditions with uncertain conditions. I will phrase many sentences structured as “if…, you need to do…”

Interviewee 8

When communicating via mobile apps, patients might be viewed differently by doctors, depending on the app selected. A notable distinction is between the 1-to-1 conversation on WeChat and communication via medical platforms. Based on the aforementioned responses of the interviewees and observations of the content of the Haodf app, we collated the disparities between these 2 communication modalities, as shown in Table 4. This table indicates that 1-to-1 messaging on WeChat is more restricted to known patients than interactions on medical platforms; in the latter case, doctors can communicate with both known and unknown patients. Medical platforms are accessible to everyone on the internet, allowing interested parties to review feedback from other patients and search for their desired doctors.

**Table 4:** Differences between 2 kinds of communication modalities.

Feature	WeChat (1-to-1 chat)	Medical platform
Patients	Acquaintances	Known and unknown patients
Trust	Based on reliable intermediaries or multiple rounds of communications	Based on cumulative feedback from patients on the platform
Outsiders allowed or not	Closed to outsiders	Open to outsiders
Boundary between personal and professional lives	The lack of boundary between personal life and professional work	Being professional
Cashing in on knowledge	Unable to cash in on one’s knowledge immediately	Able to cash in on one’s knowledge immediately

Regarding WeChat-facilitated communication, it is noteworthy that this is an enclosed mode of communication that only occurs between well-acquainted individuals, thus safeguarding the privacy of conversations. As previously discussed, the interviewees had varying degrees of willingness to share their personal accounts with others. Of the 4 (36%) interviewees using medical platforms, 3 (75%) rarely shared their WeChat accounts with patients. In China, doctor-patient communication is intertwined with social connections (*guanxi*). All interviewees were willing to share their personal WeChat accounts with patients if they were introduced by well-acquainted intermediaries, such as colleagues, relatives, or friends in hometowns or other acquaintances who could provide mutual benefits in other domains.

### Interviewees’ Views on the Hospitals

Of the 11 interviewees invited to participate in our study, 3 (27%) were department directors, thus expressing attitudes reflective of those of their affiliated hospitals. As reported by these interviewees, the hospitals have never hindered employees from using mobile apps to connect with patients; instead, they have actively implemented supportive strategies to promote online mobile services, such as making online medical appointments via their official portals. Nevertheless, such affiliation with hospitals still imposes certain constraints on doctors’ adoption of mobile apps.

The primary factor influencing doctors’ reliance on mobile phones for improved work efficiency is the multitude of tasks assigned to them. Except an interviewee from a local community clinic, all remaining interviewees reported the issue of overworking in hospitals, dedicating an average of 50 hours per week to their duties. The heavy workload prompts some doctors to use medical platforms to enhance communication efficiency with patients. Additionally, busy daytime schedules necessitate the use of WeChat for coordination tasks within departments and hospitals. Moreover, doctors in public tertiary hospitals are burdened with research and academic responsibilities, adding to their workload. Here is 2 (18%) interviewees’ feedback with regard to their typical working situations.

For every week, I have 2 surgery days, Monday and Wednesday, and I won’t be able to get off work until 9:00 p.m. or 10:00 p.m. on these days. Sometimes, it will be as late as 1:00 a.m. and 2:00 a.m. I have 2 half days scheduled for outpatients, one with around 50 patients and the other with around 35 patients. I need to work almost every day, even for the weekend. Besides, I need to have 1 night staying in the hospital in each week.

Interviewee 5

In addition to being a doctor, I am also an associate professor in a medical school… For each school year, I teach 4-5 courses to students, and I am also an advisor to 4 graduate students (master students)… I am not only in need to publish academic articles for myself in order to get higher professional titles, I also need to push my graduate students to publish articles... Using WeChat for working happens very often, and I have to tell them not to bother me after 10:00 p.m.

Interviewee 7

Despite the various tasks assigned by their affiliated hospitals, health care professionals’ compensation remains comparatively low in China. Among the interviewees working in public hospitals, more than half mentioned that their income is not satisfactory, especially compared to their counterparts in private hospitals with higher incomes. This point of view was shared by interviewee 3, who left a public hospital and joined a private dental clinic due to an unsatisfactory income. Under such circumstances, the adoption of medical platforms could increase doctors’ income, such as interviewee 6, who managed to make extra money by prescribing medications to online patients on medical platforms.

Despite the challenging tasks for interviewees in tertiary hospitals, most of them were still reluctant to leave their affiliated hospitals. For example, 2 (18%) interviewees pointed out 2 major reasons that made it unlikely for them to disconnect their affiliation with their employers: the institutional support provided by the hospitals and the hospitals’ reputation to attract more patients. As stated by interviewee 1, relying solely on medical platforms is not a feasible option for them, as it is their affiliation with hospitals that provides institutional support for their careers. Interviewee 2 emphasized the predominant role of hospitals’ reputation in attracting new patients.

Online medical platforms are operated only for profits from doctors’ work. Once the disputes between doctors and patients break out, these platforms could not do anything to protect doctors and they only turn in the online communication records between doctors and patients. Doctors could hardly count on these platforms to protect them.

Interviewee 1

In China, most people care more about hospitals than doctors. If Chinese people care more about doctors, that will be a positive impact upon doctors to build their personal reputation. However, doctors are indispensable with their hospitals in China. We cannot survive without our hospitals.

Interviewee 2

In addition to the formal affiliation, these interviewees also have strong informal connections with their employers, particularly with their colleagues within the same organization. According to the interviewees, most doctors value their connections with their colleagues and quite often receive requests from them to provide medical services to their friends. These connections serve as crucial intermediaries for new patients, helping identify the most appropriate medical expertise for specific conditions, as mentioned by interviewee 9. However, such strong ties might also become a burden for some doctors, as exemplified by interviewee 3, who chose to leave their former employer due to the perceived disturbance caused by these connections.

My colleagues often introduce patients to me. I will also introduce some patients to my colleagues. We cooperate with each other. We are very clear about the inside information with regard to which doctor is good at which disease. When we introduce some patients to other doctors, we make comprehensive evaluations on their medical skills, as well as their personality.

Interviewee 9

When I worked with a previous employer (a public tertiary hospital), the relationship between me and my colleagues was very close. We were like a family. Now my connections with my colleagues in this private clinic are straight working cooperation. My former colleagues were kind of disturbing for me. I rarely introduced my friends to other doctors, because I thought it would bring some extra burden for others. However, my former colleagues would bring many patients to me. I do not like this kind of relationship, and this is one of the reasons that I chose to leave my former employer.

Interviewee 3

## Discussion

### Principal Findings

According to the semistructured interview responses, we extracted 5 themes from the verbal data of the interviewees’ narratives. First, doctors have a variety of options for communicating with patients via mobile apps, with the predominant ones being social networking apps (eg, WeChat) and medical platforms (eg, Haodf). Second, all interviewees use WeChat to facilitate communication with patients, although their willingness to share personal accounts varies; they are more likely to do so with reliable intermediaries. Third, fewer than half of the doctors, all from tertiary hospitals, have adopted medical platforms. Fourth, preferences for in-person, WeChat, or medical platform communication reflect doctors’ views on different patient cohorts. Fifth, doctors’ choices of a particular kind of mobile app are largely affected by their affiliation with their employers, which stems from their professional duties to accomplish multiple tasks assigned by hospitals or the necessity of maintaining social connections with their colleagues.

Our findings are consistent with previous research in some aspects. Several studies have documented the use of WeChat for improving doctor-patient communication for managing certain diseases, such as advanced pancreatic ductal adenocarcinoma in China [29]. The interviewees’ reservation to share personal accounts has also been echoed in previous studies on the controversies arising from the use of personal social media for medical services [30]. Meanwhile, the finding that those adopting medical platforms are all from public tertiary hospitals is also consistent with prior results [31].

However, in contrast to previous studies, our study revealed that doctors’ choices of mobile apps are more than just their personal choice. A heavy workload, as mentioned in theme 4; comparatively low incomes; and strong social connections between colleagues, as mentioned in theme 5, demonstrate that there are strong bonds between interviewees and hospitals. These constraints were consistently observed across all interviewees from public tertiary hospitals. Previous studies [32,33] and media reports [34] have documented the popularity of these issues in China. Even though most interviewees in tertiary hospitals complain about the stressful workload and low income, few of them are willing to give up their positions in their hospitals. As discussed in theme 5, they rely on these tertiary hospitals to provide institutional support, such as attracting patients and safeguarding against potential disputes.

In terms of the particular type of mobile app to facilitate doctor-patient communication, most interviewees tend to choose the ones enabling them to complete various tasks assigned by their affiliated hospitals beyond regular working hours. In other words, their online communication with patients in mobile apps is an extension of their offline clinical service. They are bonded by not only their affiliated hospitals but also their social connections with others. Some studies from Western contexts have often highlighted doctors’ privacy concerns regarding mobile communications [11]. However, such privacy concerns were not prominent among the interviewees, who share their personal social medial accounts with certain patients.

Within these bonds, it is difficult for doctors to reach their individual goals of increasing their income by using medical platforms, such as Haodf, or having more spare time by not using any mobile apps after work. The findings provide some support for the validity of the single-triangle model, demonstrating the impact of the doctor-hospital relationship on the patient-doctor relationship. However, this model cannot explain why these bonds are so influential. Thus, the model we proposed at the beginning warrants reassessment to enhance our comprehension of doctor-patient interactions facilitated by mobile apps within China’s health care landscape.

### Remodeling the Relationships Among Doctors, Patients, and Hospitals

Behind the possible influence of hospitals are macrolevel factors. In the revised model (Multimedia Appendix 2), the triangle involving doctors, patients, and hospitals serves as a projection from a bigger triangle involving state, society, and health care provision. This double-triangle model indicates that the equilibrium within the former triangle is influenced by the dynamics of the latter triangle.

The influence of the state represents governmental influence. Governmental involvement in China’s social and health care spheres is pervasive, manifesting in regulations governing mobile businesses and the oversight of medical institutions. The most notable manifestation of the state’s involvement can be seen in the current prevailing status of public hospitals [35]. However, governmental funding for these public hospitals is insufficient to support their long-term growth [36]. Under such circumstances, these hospitals need to accommodate increasing patient loads to sustain their development. This explains why the interviewees from public tertiary hospitals are obligated to work for prolonged hours in order to accomplish various tasks assigned by their employers.

The influence of society embodies China’s social traditions, particularly its reliance on social connections for various activities. As members of this society, doctors also need to comply with these traditions. Our findings indicate a popular pattern among the interviewees to offer informal medical advice, particularly via WeChat, to patients introduced by colleagues, relatives, friends, or fellow townspeople. Previous studies suggest that social connections play a role in building patient-doctor trust in offline services [37], and our study provides evidence for trust building through communication via mobile apps.

The influence of health care provision refers to the general characteristics of China’s health care service, with 2 being particularly important. First, patients in China are not constrained by stringent referral policies, which enables them to have more flexibility in choosing their doctors [37]. Second, the patients are more inclined to go to tertiary hospitals to seek medical services, even for mild ailments [38], which is connected to the first characteristic. In this context, doctors working in those hospitals (eg, interviewees 5 and 8) are more likely to use medical platforms as a means to promote their departmental or personal reputations to attract more patients.

### Rethinking the Influence of Mobile Apps

Following the discussion of the significant role of hospitals in the doctor-patient relationship, a further question arises regarding the potential impact of mobile apps on the patient-doctor-hospital dynamic. In the extant literature, online health care, including web-based consultation, has emerged as a vital alternative solution to offline health care provision, especially in terms of addressing the flaws and deficiencies persistently embedded in offline services [17]. However, our results suggest that the impact of mobile apps might be more nuanced than we initially assumed. On the one hand, their impact on offline services might be misunderstood to some extent. Although newly emerged mobile apps are gaining increasing prominence, their potential is not strong enough to challenge the predominant position of offline services, especially in the context of China’s health care system. On the other hand, they might exert indirect impacts on those involved in doctor-patient communications, which might be neglected. Doctors could leverage mobile apps to promote their personal reputation, rather than simply relying on their institutional prominence to attract new patients.

#### Online vs Offline Health Care Provision

In our study, we found that 4 of 11 interviewees (4 of 9 doctors from tertiary hospitals) have adopted medical platforms and taken advantage of medical platforms as a more convenient means to deliver medical services to patients or to enhance their reputation to attract more potential patients for in-person hospital visits. However, it is noteworthy that offline patients remain the primary focus. For complex and severe diseases, offline services provide more advantages, as face-to-face interactions and on-site medical examinations could provide more reliable information. In a relevant study on patients’ selection of doctors from Haodf, Chen et al [39] reached a similar conclusion that online health services are well connected to offline services, especially for those departments requiring medical tests and surgeries.

Additionally, as the foremost offline service providers, hospitals have never been passive observers of emerging medical platforms but have taken a series of actions to address such challenges posed by medical information technologies. These hospitals never discourage their doctors from using medical platforms or WeChat for medical consultation purposes; rather, they are becoming more proactive in the implementation of such mobile apps to enhance the accessibility of medical services to patients [40], especially since the outbreak of COVID-19 [9]. As reflected in Table 3, almost all interviewees’ employers have created official WeChat accounts for the public to access, via which patients can make medical appointments or review their medical exam results. Alternatively, they could develop their own medical platforms with functionalities mirroring existing commercial medical platforms.

Based on our observations, an isomorphic pattern might better characterize the development of both online and offline services. The predominant role of public tertiary hospitals as the primary providers of offline services is likely to persist in the realm of online medical service provision. With the adoption of online services becoming a universal trend among all hospitals, the value of online platforms tends to mainly lie in their supportive functions.

#### Traditional Society vs Modern Mobile Technology (Individuals vs Institutions)

The choices that doctors make regarding mobile apps might be impacted by their affiliations with particular hospitals, and the future progress of online health care services might also be closely tied to these institutions. However, by facilitating interactions between doctors and patients outside conventional clinical settings, these apps provide more opportunities for doctors to communicate with patients and to promote their professional reputation.

The establishment of individual doctors’ personal reputation as competent health professionals is crucial for their career development [41]. As reported by interviewee 2, patients often rely on their employers’ reputation or doctors’ professional titles to search for an appropriate doctor, especially for the initial medical visit. For those using medical platforms, interviewee 6 (an attending doctor) diverts some offline patients to online medical platforms, suggesting that a patient tends to choose them based on their personal reputation rather than their affiliation with an esteemed hospital or professional title. With the advent of medical platforms, patients can review feedback from other patients when searching for desired doctors. As Table 4 suggests, these doctors’ medical skills and ethical values are more accessible to those outside of conventional clinical settings.

Social networking apps could also contribute to the dissemination of doctors’ professional reputation. Using 1-to-1 communication with patients via WeChat, doctors can provide convenient medical consultation to patients, or they can use group discussions to facilitate information dissemination to a wider audience. As Table 2 indicates, 6 of 11 interviewees use WeChat Moment functions, and 3 of 11 use short-term video functions, sharing the success stories of their medical treatments.

Although both kinds of apps, medical platforms and social networking apps, can facilitate doctors’ reputation, the choice of apps represents a different pattern of social interaction. As Table 4 suggests, social connections have been further used in social networking apps, while medical platforms are more reflective of modern society’s norms of personal interactions. *Guanxi* is a prominent property of traditional societies, where interpersonal trust largely relies on well-established social connections [37,42]. With the help of WeChat, the traditional interaction pattern continues in doctor-patient communication.

In contrast, medical platforms are more likely to attract the registration of new patients than social networking apps. The practice of being open to strangers on medical platforms aligns with the norms of personal interactions in modern society. Patients can easily access detailed information under doctors’ profiles to understand their professional skills and experiences, without having to rely on their friends or relatives in hospitals to obtain that information. Moreover, each doctor could maximize the utility of their professional skills by cashing in on their knowledge immediately, without having to depend on their social connections for potential benefits in the future.

In the modern era, medical platforms and social networking apps are among the most innovative creations. Through the assistance of medical platforms, patients are empowered to choose their preferred doctors by following guidelines on how to acquire detailed information about doctors’ skill sets and experiences [43]. Our study suggests that doctors are also empowered as they can expand their personal reputation beyond hospital confines.

### Limitations

Several limitations of our study should be acknowledged. First, it remains controversial whether the sample of doctors we selected for interviews was representative enough to reflect the general situation of all doctors in the medical community. Nevertheless, the qualitative research methodologies we adopted in this study did have great potential to investigate underresearched topics and population groups. This study provides meaningful implications for future studies that might consider incorporating quantitative methods to add more potent evidence in this field. Second, another issue arises due to the overwhelming number of doctors from tertiary hospitals. As suggested by previous studies, doctors from these hospitals are more inclined to using medical platforms, and residents tend to seek medical services from these hospitals even for nonsevere conditions in China. As such, the overrepresentation of doctors from these hospitals might not undermine the validity of findings from our study. However, it is highly suggested that future studies conduct more in-depth investigations to understand the distinct behavior patterns of doctors from tertiary hospitals as compared to their counterparts from community clinics. Third, the selection of Hangzhou might merit questions about the external validity of the findings. Hangzhou is the most dynamic city in terms of its acceptance of mobile apps, as mentioned in the *Methods* section. If doctors in Hangzhou have reservations about choosing a particular kind of mobile app, doctors in other cities probably will have similar concerns.

### Conclusion

In contrast to the previous studies focusing on a single type of mobile app, our study further revealed doctors’ adoption patterns across a wide range of mobile platforms, and the findings suggest their choices are aimed at improving their communication with patients, depending on the specific needs in offline services. Despite constraints posed by their affiliation with health care institutes, doctors still retain some autonomy in the adoption of medical information technologies. This autonomy enables them to leverage their medical expertise for disseminating knowledge or providing convenience to patients through social networking apps or medical platforms. The proliferation of medical information technologies offers doctors an alternative avenue to promote their own professional reputation without the necessity of relying on institutional support.

Our findings suggest that doctors’ adoption of mobile apps is affected by macrolevel factors, particularly institutional interventions stemming from hospital affiliations. The presence of a state-society-health care provision nexus plays a pivotal role in shaping the doctor-patient-hospital relationship. Such findings also provide meaningful implications to inform doctors’ adoption behavior in other countries, especially lower- and middle-income countries, which are confronted with similar issues of transitioning from traditional to modern societies. By addressing these macrolevel constraints, strategies can be devised to enhance the quality and efficiency of medical service delivery through mobile apps.

## Appendix

Multimedia Appendix 1 Semistructured interview questions.

Multimedia Appendix 2 Double-triangle model to understand mobile communication between doctors and patients.

## Abbreviations

COREQ: Consolidated Criteria for Reporting Qualitative Research
GDP: Gross Domestic Product
mHealth: mobile health
RMB: Renminbi
PDAs: Personal Digital Assistants
WHO: World Health Organization
