# Supplementary material for: Doctors’ Personal Preference and Adoption of Mobile Apps to Communicate with Patients in China: Qualitative Study
Source: JMIR Mhealth Uhealth. 2024 Jun 10;12:e49040. doi: 10.2196/49040 (PMC11196915; doi:10.2196/49040)
Supplement: Multimedia Appendix 1 [file mhealth_v12i1e49040_app1.docx]

**Appendix 1.** Semi-structured interview questions

| Interview questions |
| --- |
| How many hours do you work in a working day? How many hours in a week? What kind of specific tasks do you need to have? |
| Will your colleagues or your patients bother you during your off-duty time? |
| Have you contacted your patients via WeChat? Are you willing to share your WeChat account with your patients? |
| Do your feel that communications directly with patients or indirectly via the intermediary have increased after the expansion use of mobile phones? Do others share your WeChat account with other potential patients? If so, who are they? |
| Do hospitals restrain or encourage doctors’involvement in medical platform? |
| Do hospitals have their own medical platforms? |
| How do you choose a particular medical platform from a variety of choices? |
| Will you online communication with patients affect your offline communication with them? What are the differences between online and offline communications? |
