# Supplementary material for: Doctors’ Personal Preference and Adoption of Mobile Apps to Communicate with Patients in China: Qualitative Study
Source: JMIR Mhealth Uhealth. 2024 Jun 10;12:e49040. doi: 10.2196/49040 (PMC11196915; doi:10.2196/49040)

**Appendix 2.** Double-triangle model to understand mobile communication between doctors and patients.


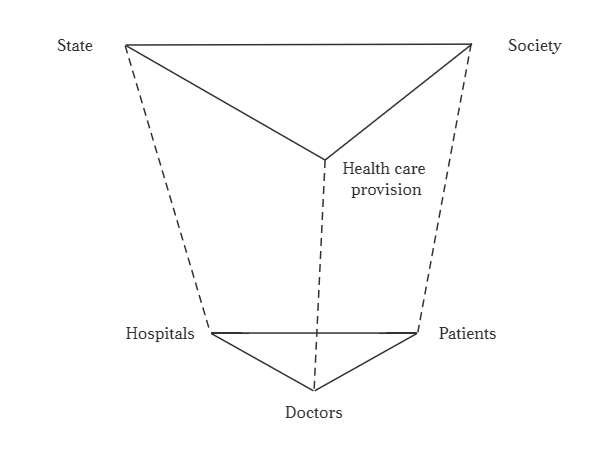

Supplement: Multimedia Appendix 2 [file mhealth_v12i1e49040_app2.docx]
